# Insights into the Antiviral Immunity against Grass Carp (*Ctenopharyngodon idella*) Reovirus (GCRV) in Grass Carp

**DOI:** 10.1155/2015/670437

**Published:** 2015-02-09

**Authors:** Youliang Rao, Jianguo Su

**Affiliations:** ^1^College of Animal Science and Technology, Northwest A&F University, Yangling 712100, China; ^2^College of Fisheries, Huazhong Agricultural University, Wuhan 430070, China; ^3^Freshwater Aquaculture Collaborative Innovation Center of Hubei Province, Wuhan 430070, China

## Abstract

Global fish production from aquaculture has rapidly grown over the past decades, and grass carp shares the largest portion. However, hemorrhagic disease caused by grass carp reovirus (GCRV) results in tremendous loss of grass carp (*Ctenopharyngodon idella*) industry. During the past years, development of molecular biology and cellular biology technologies has promoted significant advances in the understanding of the pathogen and the immune system. Immunoprophylaxis based on stimulation of the immune system of fish has also got some achievements. In this review, authors summarize the recent progresses in basic researches on GCRV; viral nucleic acid sensors, high-mobility group box proteins (HMGBs); pattern recognition receptors (PRRs), Toll-like receptors (TLRs) and retinoic acid inducible gene I- (RIG-I-) like receptors (RLRs); antiviral immune responses induced by PRRs-mediated signaling cascades of type I interferon (IFN-I) and IFN-stimulated genes (ISGs) activation. The present review also notices the potential applications of molecule genetic markers. Additionally, authors discuss the current preventive and therapeutic strategies (vaccines, RNAi, and prevention medicine) and highlight the importance of innate immunity in long term control for grass carp hemorrhagic disease.

## 1. Introduction

Over the past three decades, aquaculture industry has made impressive progress and constituted high quality protein for much of the world's population. In China, aquaculture industry has become a major power to promote sustainable, rapid, and stable development of China's fishery [1]. Grass carp (*Ctenopharyngodon idella*) is an important freshwater economic fish in China, and its production accounts for 18.10% of total freshwater fishery in 2013, which is also the largest production of fish in the world. However, hemorrhagic disease caused by grass carp reovirus (GCRV) seriously affects the grass carp cultivation industry.

Fish innate immunity plays an essential role in protecting host against invading pathogens [2]. Similar to mammals, fish possess evolutionary conserved pattern recognition receptors (PRRs) that are responsible for sensing the presence of pathogen-associated molecular patterns (PAMPs) which are structurally conserved among many microorganisms [2, 3]. PRRs can be divided into four different classes: Toll-like receptors (TLRs), retinoic acid-inducible gene (RIG) I-like receptors (RLRs), NOD-like receptors (NLRs), and C-type lectin receptors (CLRs) [3]. Among these PRRs, TLRs and RLRs play important role in recognition of viruses or viral PAMPs [3, 4]. Upon activation by viral components, TLRs or RLRs transmit signals to the downstream adaptor molecules, which induce a large scale amplification of signaling cascade to activate interferons (IFNs) or nuclear factor-*κ*B (NF-*κ*B) pathways via IFN regulatory factors (IRFs) [5, 6]. Subsequently, IFN-I along with IFN-stimulated genes (ISGs) mediated the first antiviral defense [7].

Over the past decades, to propose the effective prevention or therapeutic strategy for hemorrhagic disease, a great attempt focused on the understanding of the pathogenesis of GCRV, pathogen recognition, and revealing the regulation mechanism of antiviral immune network in teleosts. This review summarizes the recent progresses on GCRV, antiviral signaling pathway in teleosts and the achievement in prevention of hemorrhagic disease. We highlight the importance of molecular breeding based on the understanding of innate immunity in the developing of disease theoretical strategies.

## 2. Pathogen

GCRV, a member of genus* Aquareovirus* in the family Reoviridae, was the first viral pathogen to be identified from aquatic animals in China in 1983 [8]. The virus is a nonenveloped icosahedral spherical in appearance with 5 : 3 : 2 three-dimensional symmetry, which is comprising 11 double-stranded RNA genome segments surrounded by multiple concentric protein capsids. The 11 genome segments encode seven structural proteins (VP1–VP7) and five nonstructural proteins. The core layer is composed of 5 proteins (including VP1–VP4 and VP6), while the outer capsid of GCRV contains 200 trimers formed by VP5-VP7 heterodimers [9, 10]. So far, more than 20 strains of GCRV have been reported, and ten GCRV strains have complete genome sequences (Table 1), which are GCRV-873, AGCRV [11], GCRV-HZ08 [8], GCRV-HuNan794, GCRV-106, GCRV-GD108 [12], GCRV-918, GCRV-HeNan988, HGDRV (formerly GCRV-104) [13], and GCReV-109 [14]. Based on VP6 sequences, the known GCRV stains (isolated in China) can be clustered into three groups, with representative isolates GCRV-873 (group I), GCRV-HZ08 (group II), and HGDRV (group III) [8]. However, Pei et al. classified GCRV-HZ08 into group I, GCRV-873 into group II, and HGDRV (GCRV104) into group III [14]. When AGCRV is introduced to the cluster, the GCRV strains are divided into four groups [15]. However, there is subtle difference of the phylogenetic relationships of GCRV when VP4, VP6, and VP7 are used for the clusters, respectively [15]. Although different people have different views on the cluster, one point that GCRV-873, GCRV-HZ08, and HGDRV are the representative strains of three different groups is consistent [8, 14, 15]. With the sequence distinctions among the strains, the cell culture characteristic, virulence, pathogenesis, and antigenicity of each strain are diverse. For instance, GCRV-097 strain can induce significant cytopathic effects (CPE) of* C. idella* kidney (CIK) cells, massive abdominal hemolysis and obvious haemorrhage in muscle, skin, intestine, and gill of grass carp, resulting in a high mortality rate of grass carp [16–19]. As a novel fish reovirus, HGDRV can proliferate and induce significant apoptosis or CPE in CIK cells [13, 20]. Similarly, GCRV096 can also cause the CPE in CIK when the cells are infected [15]. GCRV-873, the first strain isolated from hemorrhagic grass carp in China, can form CPE in CIK cells but lose the ability to infect grass carp [21, 22]. In contrast, GCRV-861 infection causes high mortality rate of grass carp and rare gudgeon (*Gobiocypris rarus*) but fails to cause CPE in CIK cells [23]. GCRV-991 which shows much similarity with GCRV-873 in protein molecular weights and antigen properties possesses strong pathogenicity to grass carp and causes obvious CPE in CIK cells [22, 24]. However, GCReV-109, CGRV-HZ08, and GCRV-GD108 cannot induce CEP [14, 25]. Both GCRV-JX01 and GCRV-JX02, isolated from the same diseased grass carp sample, can produce progeny in CIK cells, but only GCRV-JX01 induced CPE in infected cells [26].

## 3. Innate Antiviral Immunity

### 3.1. HMGBs

High-mobility group box proteins (HMGBs), a newly discovered family of nucleic acid sensors, play important role in the signal-transducing antiviral immune response [28–30]. HMGBs are highly conserved chromatin-associated proteins from invertebrate to vertebrate [19, 31–37]. In mammals, HMGBs contain four family members: HMGB1, HMGB2, HMGB3, and HMGB4 [38]. However, the members of HMGBs in some low vertebrates or invertebrates are various. For example, two paralogs of mammalian HMGB1, HMGB2, and HMGB3 are present in some teleosts: zebrafish (*Danio rerio*), salmon (*Oncorhynchus*), carp (*Cyprinus carpio*), and grass carp [30, 34]; but no HMGB3 subfamily is present in* Tetraodon*, stickleback, medaka (*Oryzias latipes*), and fugu (*Takifugu rubripes*), even though two paralogous of HMGB1 and HMGB2 were detected [34]; in* Litopenaeus vannamei*, HMGBs have two member: HMGBa and HMGBb [39].

Evidences have highlighted that HMGBs function as universal sentinels of nucleic-acid-mediated innate immune responses [28]. On one hand, they promiscuously recognize immunogenic nucleic acids and then initiate immune response by transducing signals to PRRs, such as TLRs, RLRs, and other cytosolic receptors [29]. On the other hand, they suppress the innate immune responses by binding nonimmunogenic nucleotides [40]. In mammals, HMGB1 and HMGB3 bind both DNA and RNA, but HMGB2 only binds to immunogenic DNA, not RNA [28]. In grass carp, all the HMGBs family members can respond to synthetic dsRNA (poly(I:C)) and GCRV challenge. Overexpression of grass carp HMGBs significantly delays the GCRV-induced CPE in CIK cells [19, 31, 32]. Meanwhile, the replication of GCRV in HMGBs-overexpressing CIK cells is remarkably inhibited [19, 31]. In all the HMGBs overexpression cells, transcription levels of some vital downstream molecules of TLRs and RLRs are notably modulated, especially for some adaptor molecules: Toll/interleukin-1 receptor (TIR) domain containing adapter inducing IFN-*β* (TRIF, also known as TICAM1), IFN-*β* promoter stimulator-1 (IPS-1), and myeloid differentiation factor 88 (MyD88). HMGBs overexpressions induce the upregulation of these genes. It is well known that MyD88 and TRIF are two important adaptor proteins of TLRs pathway and IPS-1 is responsible for the signal translation of RIG-I and MDA5 [5, 41, 42]. So signals of HMGBs may transmit to the downstream proteins via TLRs and RLRs.

Generally, HMGBs are typical nuclear proteins, while pathogeny challenge can induce the proteins shuttling from nucleus to cytoplasm and further secreting to the extracellular medium [43–45]. Like mammalian HMGBs (1–3), teleosts HMGBs compose of two basic HMG box domains and an acidic tail [30, 34, 36, 46]. Nuclear localization signals (NLSs) in the HMG box determine the nuclear localization of the proteins and the acidic tail contributes to nuclear localization [38, 47, 48]. Under basal conditions, all the six grass carp HMGBs exclusively localized to the nucleus in CIK cells, while virus invasion or pathogenic stimuli induce the nucleocytoplasmic translocation of HMGBs to various degrees [30]. Truncated and chimeric domain experiments demonstrated that the N-terminal domain confers nuclear localization, but the nucleocytoplasmic migration of HMGBs attributes to the dynamic balance or intercellular interaction between the HMG box domain and acidic tail domain. For the six HMGBs of grass carp, CiHMGB2a and CiHMGB3b rarely shuttle from nucleus to cytoplasm in response to GCRV, poly(I:C), and LPS challenge. The ratio ranking of other members nucleocytoplasmic translocation in response to GCRV infection was CiHMGB2b > CiHMGB1a > CiHMGB3a > CiHMGB1b; poly(I:C) also induces the relocations of CiHMGB1a, CiHMGB1b, and CiHMGB3a; LPS stimulation only notably evokes the robustly nucleocytoplasmic shuttling of CiHMGB1b [30]. These results imply various roles of HMGBs in response to pathogenic challenges.

**Figure 1:**
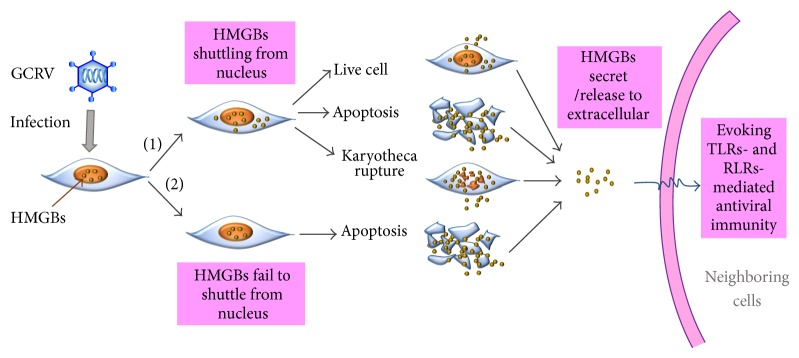
Antiviral immune response of HMGBs induced by GCRV infection in CIK cells. GCRV infection induces diverse nucleocytoplasmic shuttling of grass carp HMGBs via two main methods: (1) upon GCRV infection, some HMGBs such as HMGB1a, HMGB1b, HMGB2b, and HMGB3a shuttle from nucleus to cytoplasm. Subsequently, a huge member of cells subject to apoptosis or karyotheca rupture, which result in cells death and passive release of HMGBs. On the other hand, some live cells can also actively secrete HMGBs to extracellular space; (2) even though GCRV fail to evoke nuclear exports of some HMGBs such as HMGB2a and HMGB3b, but the cells will undergo necrosis or damage. So those HMGBs are released to the extracellular matrix. Afterwards, the extracellular HMGBs initiate activation of TLRs- and RLRs-mediated antiviral immunity of neighboring cells.

HMGBs can be actively secreted from innate immune cells or passively released from dead or injured cells [29, 45]. GCRV infection evokes active secretion of some HMGB members that are easy to shuttle from nucleus and passive release which is associated with necrosis and death of all HMGBs (Figure 1); extracellular presence of all the six HMGBs is detected by western blotting [30]. In infected cells, GCRV replicates and assembles in cytoplasm, where they form specific structures termed virus inclusion bodies (VIBs) that separate viral particles from the adjacent cytoplasm [9]. Hence, cytoplasmic occurrence of HMGBs may be responsible for recognition of virus-derived nucleic acid and transmit signal to cytoplasmic antiviral receptors. Extracellular HMGBs may exert their cytokine-like or proinflammatory function via interaction with the cell surface receptors of neighboring cells (Figure 1). To date, with the regression of antibodies production in fish, especially for commercial fishes, the researches mainly rest on transcription levels. More studies are urgent to uncover the regulation mechanism of these viral nucleotide sensors in the signal transduction.

### 3.2. TLRs Signaling Pathway

Typically, the innate immune system recognizes pathogen invasion via a variety of PRRs. During the past decades, study on PRRs has expanded rapidly, and massive amounts of scientific evidence attest to their importance in innate immunity. TLRs are the earliest characterized and the most extensively studied PRRs in both vertebrates and invertebrates [49]. Up to now, thirteen TLRs (TLR1–13) have been identified in mammals. In teleost, at least 19 TLR types (1, 2, 3, 4, 5, 7, 8, 9, 11, 14, 18, 19, 20, 21, 22, 23, 25, 26, and 27) were discovered, except for the paralogous or duplicated members of TLRs; TLR6 and TLR10 are absent from all fish genomes sequences to date [2, 6, 49, 50].

In teleosts and mammals, TLRs are characterized by three domains: the N-terminal leucine-rich repeat (LRR) domain, a Toll/interleukin-I receptor domain (TIR), and a transmembrane domain (TM). LRR domain is the functional domains to play an important role in the recognition of PAMPs [2]; TIR domains are responsible for activating downstream signaling by interaction and recruitment of various adaptor proteins [41, 51]. Unlike the cytoplasmic receptors, TLRs mainly locate at cell surface and endosomal compartments [42]. Mammalian TLR1, TLR2, TLR4, TLR5, TLR6, and TLR11 are expressed exclusively on the cell surface and recognize molecules derived from microbes, while TLR3, TLR7, TLR8, and TLR9 are expressed in some intracellular vesicles such as endoplasmic reticulum (ER), endosomes, multivesicular bodies, and lysosomes [41, 42, 52]. Under normal conditions, the intercellular TLRs are ER resident whereas they translocate to the endosome via the common secretory pathway by traversing the Golgi upon activation, and some chaperone proteins are required for this efficient translocation [42].

**Figure 2:**
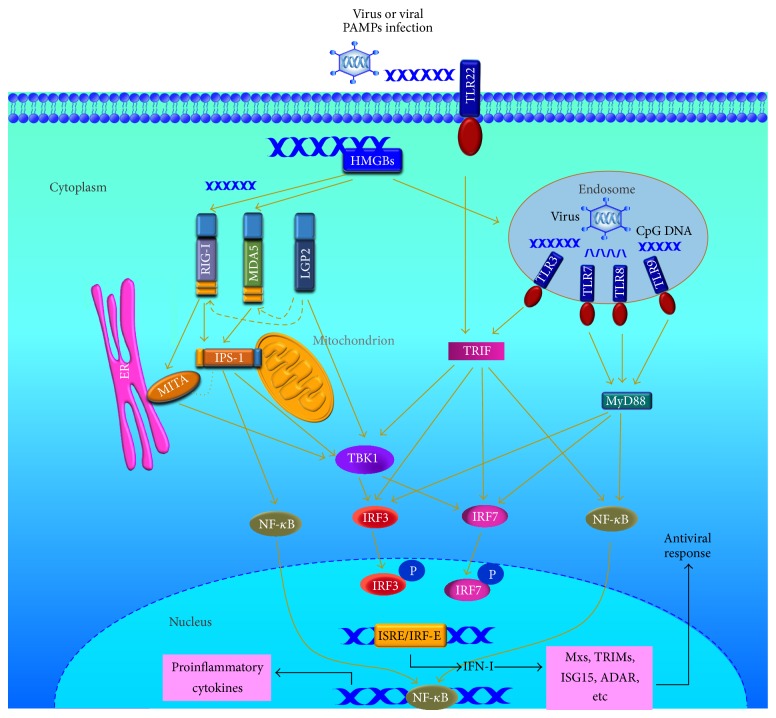
Schematic overview of intracellular antiviral immune signaling in teleosts. Fishes have conserved intercellular PRRs to sense virus or viral PAMPs. TLR3 and TLR22 sense dsRNA and transmit signals to the downstream adaptor IRIF; endosome localized TLR7/8 and TLR9 recognize ssRNA and CpG DNA, respectively, and then deliver signal to MyD88. Like mammalian RLRs, RIG-I and MDA5 recognize dsRNA or ssRNA in different length and activate mitochondrion IPS-1. With no CARD domain, LGP2 is thought to fail to interact with IPS-1 but can transmit signal to TBK1. Studies also indicate that LGP2 can mediate signals of RIG-I and MDA5. Fish MITA localizes in ER, but it is in close vicinity with IPS-1 in mitochondrial-ER contact regions. MITA participates in antiviral activation of IFN or ISGs downstream of RIG-I and MDA5 through MITA-TBK1-IRF3 pathway. TRIF and IPS-1 transfer signal through NF-*κ*B and TBK1-IRF3/7-IFN-I pathway. MyD88 signal activates IRF3/7 and NF-*κ*B, not TBK1. Upon phosphorylation, IRF3 and IRF7 transmit to nucleus and induce the production of IFN-I which induces antiviral immune response along with the activated ISGs. The activated NF-*κ*B also transmits to nucleus initiating the activation of proinflammatory cytokines. Cytoplasmic HMGBs can promiscuously sense immunogenic nucleic acid and delivery to the discriminative sensors: TLRs and RLRs.

So far, more TLRs have been discovered in teleost fish and the viral PAMPs induced signaling pathways are preliminary revealed (Figure 2). Upon activation by viral PAMPs, TLRs transmit signals to their adaptor molecules which initiate the activation of NF-*κ*B and IFN-I pathways [4, 51, 53]. There are five TIR domain-containing adaptors including MyD88, TRIF, TIRAP/Mal, TRIF-related adaptor molecule (TRAM), and Sterile-alpha and Armadillo motif-containing protein (SARM) [3]. Mammalian MyD88 is required by all TLRs except for TLR3, and TRIF is used by TLR3 and TLR4 to activate NF-*κ*B, IRF3, and the production of IFN-I; TIRAP acts as an additional adaptor of TLR2 and TLR4 to recruit MyD88; TRAM serves as a bridge between TRIF and TLR4; SARM is believed to negatively regulate signaling of TRIF-dependent signaling of TLR3 and TLR4 [3, 54], while all the TLRs transmit adaptor signals roughly through two main pathways depending on the adaptors MyD88 and TRIF [2, 51]. In teleost, TRIF is required for the signaling cascade of TLR3 and TLR22, while MyD88 is essential for the downstream signaling of various TLRs, with exception of TLR3 and TLR22 (Figure 2) [51, 55].

#### 3.2.1. TRIF-Dependent Pathway

Mammalian TLR3 recognizes extracellular and intracellular viral dsRNA and initiates signaling cascades leading to NF-*κ*B activation and IFN production [3, 56]. However, both fish TLR3 and TLR22 are the receptors to sense viral dsRNA. Fugu TLR3 recognizes short dsRNA in ER and TLR22 recognizes long dsRNA on the cell surface. Both TLR3 and TLR22 induce the activation of IFN via TRIF (Figure 2) [55]. Probably, fish TLR22 may be a functional substitute for human cells surface TLR3 for detecting dsRNA virus infection [57]. Expression of grass carp TLR3 and TLR22 is also induced by poly(I:C) or GCRV challenge [58, 59]. GCRV infection increases the expression of rare minnow (*Gobiocypris rarus*) TLR3 along with its splice variant [60]. In Japanese flounder (*Paralichthys olivaceus*), TLR3 is upregulated by poly(I:C) and viral hemorrhagic septicemia virus (VHSV) (ssRNA virus) challenge, and intracellular poly(I:C) induces the expression of ISG and activity of NF-*κ*B [56]. Zebrafish TLR3 is proposed to activate NF-*κ*B and its expression level is upregulated by snakehead rhabdovirus (SHRV) infection [61]. Further studies indicate that TRIF also participates in the activation of IFN and NF-*κ*B signal pathways: zebrafish TRIF activates IFN by the interaction of TRIF with TLR3 and TANK-binding kinase 1 (TBK1), and the NF-*κ*B activation is dependent upon its interaction with receptor-interacting protein 1 (RIP1) [62, 63]. Crucian carp TBK1 interacts with IRF3 which activates IFN promoter [64]. In grass carp, TRIF overexpression induces the upregulation of IRF7 and IFN-I and significant antiviral response to GCRV infection [65].

#### 3.2.2. MyD88-Dependent Pathway

Among the teleost TLRs, TLR1 has been cloned in some fish species such as orange-spotted grouper (*Epinephelus coioides*), zebrafish, and large yellow croaker (*Pseudosciaena crocea*), and its expression is increased upon LPS and poly(I:C) stimulation or* Vibrio alginolyticus* and* Mycobacterium marinum* infection [66–68]. In mammals, TLR2 and TLR4 sense bacterial components. For example, TLR2 recognizes PGN, LAM, and triacyl lipopeptides; and TLR4 senses LPS [41]. However, the function of these two TLRs in teleost differs from that of mammals: TLR2 is proposed to recognize viral rather than bacterial ligands; and TLR4 does sense bacterial LPS [69–71]. Many fish species express two TLR5: a membrane TLR5 (mTLR5) and a soluble TLR5 (sTLR5), and they sense bacterial flagellin [69, 72–74]. Fish TLR9 senses viral and bacterial DNA. The LRR sites in TLR9 molecule of teleost can sense a variety of CpG-oligodeoxynucleotides (CpG ODNs) motifs present in different bacteria [75]; Atlantic salmon (*Salmo salar*) TLR9 interacts with synthetic ODN via a CpG-independent but pH-dependent mechanism [76]; recent study highlights that TLR9 and TLR21 cooperatively mediate activity of CpG-ODNs in zebrafish [77].

TLR7 and TLR8 recognize ssRNA and also the response to dsRNA or poly(I:C) [4, 69]. Upon GCRV infection, expression of grass carp TLR7 is upregulated in spleen but inhibited in hepatopancreas; poly(I:C) also increases the mRNA level of TLR7 in CIK cells [78]. Grass carp TLR8 is upregulated in spleen and head kidney by GCRV infection, while the transcription level is downregulated by poly(I:C) stimulation; meanwhile, in TLR8 knockdown CIK cells, the replication of GCRV is significantly inhibited [79]. Mammalian TLR7, TLR8, and TLR9 transmit signals through MyD88-dependent pathway: TLR7/8/9-mediated IFN*α* production requires the interaction between MyD88 and IRF7, which results in the activation of IFN*α*-dependent promoters. Meanwhile, the activated IRF7 translocates to nucleus and activates IFN*α* and ISGs (Figure 2) [80]. Full-length MyD88 has been cloned from grass carp and the expression level is upregulated by GCRV infection or poly(I:C) stimulation [81]. Zebrafish MyD88 transfers the signal from TLRs to downstream molecules, inducing the activation of NF-*κ*B and human IFN*β* promoters [82]. Salmonid MyD88 is also found to activate NF-*κ*B [83]. Further study indicates that MyD88 interacts with IRF3 and IRF7 modulating the IRF-induced IFN response in Atlantic salmon [84]. Recently, SARM1 and its two splice variants were identified in grass carp, and they were proved to inhibit GCRV-triggered IFN-I response by affecting the expression of TRIF, MyD88 or IPS-1, or the downstream genes [85], which indicates that SARM may function as an important inhibitor in TLR or RLR pathways.

### 3.3. RLRs Signaling Pathway

#### 3.3.1. Activation of RLRs

RLRs are a family of cytoplasmic PRRs that sense viral PAMPs in both teleost and mammals [3–5, 52]. RLR family consists of three members: RIG-I (also called DDX58), melanoma differentiation-associated gene 5 (MDA5 or IFIN1), and laboratory of genetic and physiology 2 (LGP2, also named DHX5) [2]. Similar to the mammals, teleost RIG-I, MDA5, and LGP2 contain three domains: two tandem caspase-associated and recruitment domains (CARDs) which present in the N-terminal of RIG-I and MDA5 but not in LGP2; a central DExD box helicase/ATPase domain (DExD/H) (consisting of two RecA-like helicase domains, Hel1 and Hel2, and an insert domain, Hel2i); a C-terminal repressor domain (RD, also called C-terminal domain (CTD)) [2, 3, 18, 86]. The CARDs of RIG-I and MDA5 physically interact with the CARD of IFN-*β* promoter stimulator-1 (IPS-1, alternatively called MAVS, VISA, or Cardif), the adaptor protein of RLRs, to activate the downstream signaling cascade [87]. Besides the N-terminal CARD domain, IPS-1 also possesses a proline-rich region and a C-terminal mitochondrial TM domain [88]. Lacking the N-terminal CARDs, LGP2 is unable to interact with the CARD of IPS-1 [2, 87, 89]. However, the interaction between LGP2 and IPS-1 is demonstrated in HEK 293T cells, which requires the C-terminal TM domain and the intermediate domain (residues between 300 and 444) of IPS-1 [90].

Viral infection in the cytoplasm is primarily detected by the RLRs. Studies indicate that RIG-I preferentially binds to relatively short 5′-phosphorylated dsRNA, while MDA5 binds to long dsRNA [86, 91]. Several crystal structures unveil the mechanism of RIG-I and MDA5 in the regulation of RNA recognition and triggering downstream signaling: under resting state, RIG-I exists an autoinhibited conformation: both the CARDs link to one another in a head-to-tail manner, and CARD2 form contacts with the Hel2i domain, which shields the CARDs-CARDs interaction between RIG-I and IPS-1, thereby interdicting the signal transduction [86, 92]. Upon viral infection, a structural zinc ion and a positively charged cleft-like structure within the CTD domain recognize the 5′-PPP extremity of the blunt-end base-paired RNA; the helicase domain binds to the sugar-phosphate backbone of duplexed-RNA, which results in the release of CARDs [5, 93]. Although MDA5 has a similar domain architecture as RIG-I, it recognizes dsRNA in a different manner with RIG-I. In the absence of ligand, MDA5 adopts an open conformation rather than forming interaction between CARDs and helicase domains [94]. By dsRNA stimulation, the helicase domain of MDA5 wraps around the phosphate backbone of dsRNA. Instead of the closed O-ring-like structure as in RIG-I, the MDA5 CTD is rotated by 20° to form a C-shaped ring [95]. As for LGP2, it senses signaling in response to viral stimuli by modulating the RIG-I and MDA5 signals [96–98]. Although evidences have unveiled the negative or positive role of LGP2 in the regulating of antiviral immunity, the mechanism of LGP2 mediated opposing roles is currently unclear [87, 99].

#### 3.3.2. Modulation of RLRs Signaling in Teleosts

RLRs are structural conserved proteins from mammal to teleost species, while the sequence identities within the three members are not very high [2]. Up to now, RIG-I, MDA5, and LGP2 have been identified in numerous fish species, in which LGP2 and MDA5 are common to all fish genomes, but RIG-I appears to be lost in some species [2, 87]. In grass carp, genome of RIG-I, MDA5, and LGP2 has been identified [100–102], and the expression levels of these genes were significantly upregulated in spleen and liver after GCRV infection [103–105]. By GCRV and poly(I:C) challenge, transcription level of RIG-I is upregulated in CIK cells [104]. CPE assay and viral titter reveal the significant antiviral activity of full-length RIG-I in response to GCRV infection, in which CARDs domains play a positive role but the RD domain exhibits a negative effect in the signaling channel [18]. In channel catfish ovarian cells, channel catfish virus infection significantly increases the expression of RIG-I, MDA5, and LGP2 [106]. In Japanese flounder, both MDA5 and LGP2 but not RIG-I, existed [2]; overexpression of MDA5 and LGP2 along with their adaptor IPS-1 displays remarkably antiviral activity against ssRNA (HIRRV, VHSV) or dsRNA virus (IPNV) and significantly enhances the expression of IFN, Mx, and ISG15 [89, 107, 108]. Rainbow trout (*Oncorhynchus mykiss*) MDA5 and the full-length LGP2 can protect rainbow trout gonad 2 (RTG-2) cells against VHSV; however, the LGP2 variant exerts a negative role [109]. Crucian carp LGP2 is also found as a negative regulator of both RIG-I and MDA5 in cytosolic dsRNA-induced signaling [64]. But grass carp LGP2 may act as a positive role molecule in anti-GCRV innate immune [110].

Activations of RIG-I and MDA5 induce downstream signaling by binding to IPS-1 that subsequently recruits and activates cytosolic kinases TBK1 and I*κ*B kinase complex (IKK*α*/*β*/*γ*), which are responsible for the activation of IRF3/7 and NF-*κ*B (Figure 2); then these transcription factors translocate to the nucleus and coordinate the expression of IFN-*α* and IFN-*β* [5, 51, 111]. Mammalian IPS-1 mediates the activation of NF-*κ*B and IRF3 inducing the expression of IFN-I [88]. In teleosts, zebrafish MDA5 and its two spliced shorter forms (MDA5a and MDA5b) significantly induced the activation of IFN-I promoter in response to spring viraemia of carp virus (SVCV) infection; meanwhile, the spliced form MDA5b can enhance IPS-1-induced IFN-I promoter activity [112]. Study also reveals the cooperative effects of zebrafish IPS-1 variants and RIG-I in inducing of downstream antiviral genes [113]. Atlantic salmon IPS-1 participates in the activation of IFN and NF-*κ*B upon recognition of viral dsRNA [114]; overexpression of Japanese flounder IPS-1 delays the appearance of CPE in response to HIRRV and VHSV and induces the increase of IRF3, Myxovirus resistance 1 (Mx1), and ISG15 [108]; mRNA level of IPS-1 is upregulated by GCRV infection in grass carp [115]. TBK1, a member of I*κ*B kinase (IKK) family, which associates with TRIF and IPS-1, then induces the phosphorylation of IRF3/7 [5, 116]. In grass carp, overexpression of TBK1 induces the expression of IRF7, IFN-I, and Mx1 and inhibits the replication of GCRV in CIK cells [117].

Besides IPS-1, other junction adaptor molecule, mediator of IRF3 activation (MITA, also known as STING, ERIS and MYPS), is also involved in the activation of RLRs signaling [51, 118]. Mammalian MITA interacts with IPS-1 triggering IFN-I induction via recruited TBK1 and IRF3 [119, 120]. Fish MITA localizes in ER, and MITA activates antiviral IFN or ISGs response downstream of RIG-I and MDA5 through MITA-TBK1-IRF3 pathway [64, 118]. Overexpression of grass carp MITA can upregulate the mRNA level of TBK1, IRF3, and IRF7 [121]. Studies also indicate that MITA and IPS-1 closely localize in mitochondrial-ER contact regions, but no evidence certifies whether MITA can interact with IPS-1 and RIG-I-IPS-1 complex [118].

### 3.4. NLRs

NLRs are a family of cytoplasmic PRR, which are characterized by three domains, an N-terminal protein interaction domain, a central nucleotide-binding domain (NOD, also known as NACHT domain), and a C-terminal LRR [52, 122]. The NACHT domain is responsible for nucleotide binding and self-oligomerization; the LRRs mediate pathogen sensing; and the N-terminal domain is required for the protein-protein interactions for initiating downstream signaling, by which NLRs are categorized into five subfamilies: NLRA (containing an acidic transactivation domain), NLRB (containing a baculovirus inhibitor apoptosis protein repeat), NLRC (containing CARD domains), NLRP (containing a Pyrin domain), and NLRX (containing an unknown domain) [52, 123]. Generally, NLRs recognize bacterial PAMPs such as PGN, LPS, LTAs, and MDP [52]. However, further studies imply that some NLRs participate in the regulation of antiviral immunity pathways. The mitochondria localized NLRX1 interacts with IPS-1 to modulate virus-induced INF-*β* production, which indicates that NLRX1 is a therapeutic target for enhancing antiviral responses [124].

Teleosts have conservative and abundant NLR molecules [125]. NLRs have been reported in some fish species, some of which have been proposed to certify the antiviral involvement of NLRs [122, 126]. In grass carp, GCRV infection or poly(I:C) challenge significantly unregulated expression of NOD1 and NOD2 in spleen and trunk kidney [126, 127]. Class II, major histocompatibility complex, transactivator (CIITA), a member of NLR family, was upregulated in catfish head kidney and liver but reduced in spleen post channel catfish hemorrhage reovirus (CCRV) infection [122]. All these results imply that some fish NLRs may also function as cytoplasmic PRRs in sensing virus or viral PAMPs challenge.

### 3.5. IRFs

IRFs are a large family of transcription factors involved in host immune response and regulation of IFN or ISG induction. All IRFs possess a unique “tryptophan cluster” DNA-binding domain (DBD), which is responsible for binding to the IFN promoter [128]. IRFs include 9 members in mammals, 10 members in birds, and 11 members in fish, and many IRF members are essential regulators in PRR-mediated signaling [116, 129]. Among all the IRFs, IRF3 and IRF7 are the key regulators of IFN-I expression upon viral infection. By virus infection, activations of some certain PRRs such as TLR3, RIG-I and MDA5 result in the phosphorylation and nuclear translocation of IRF3 and IRF7 [129]. In fish, antiviral effects of IRF1 has been indicated in Atlantic salmon and Japanese flounder [130], IRF3 and IRF7 in rainbow trout, large yellow croaker, crucian carp, and carp [116, 131–133], IRF5 in zebrafish [134], and IRF10 in Japanese flounder [135]. Meanwhile, zebrafish IRF10 is found to be a negative regulator to balance the innate antiviral immune response [136]. After GCRV infection, IRF5 was upregulated in spleen and head kidney, and IRF3 and IRF7 were upregulated in TBK1 and MITA overexpressed CIK cells [117, 121, 137].

### 3.6. Host Antiviral State Induced by IFN Signals

#### 3.6.1. IFN Signal in Teleosts

IFN response, the key components of innate immune, is the first line of host defense against virus infection. Mammalian IFNs have been designated into three groups: type I IFNs, type II IFNs, and type III IFNs [138]. Study indicates that fish IFNs belong to the IFN-I [51, 139]. In grass carp, transcripts of IFN-I is significantly upregulated by GCRV infection in head kidney, spleen, and gill tissues [140]. Similar to mammals, fish IFN antiviral response is initiated through the pattern recognition of virus component by TLRs and RLRs [141]. Signaling from TLRs and RLRs pathways is transmitted to IRFs and induces phosphorylation of IRFs, which translocate from cytoplasm to nucleus where they turn on IFN gene transcription by binding to ISRE/IRF-E motifs present in IFN promoters [2, 51]. In crucian carp, this early phase of IFN expression induces the expression of ISGs via JAK-STAT signaling pathway which also triggers the expression of IFN-I in turn [51]. Recently, a magnitude of ISGs has been identified in fish such as Mx, ISGs, PKR, PKZ, Gig, viperin, Drel, and TRIMs [141, 142].

#### 3.6.2. Antiviral Activation of ISGs in Teleosts

*(1) Mx.* Mx proteins that belong to the dynamin superfamily of GTPases exhibit essential antiviral activity against a wide range of viruses. Mx is composed of three domains: an N-terminal dynamin domain (containing dynamin family signature and tripartite GTP-binding motifs, DYNc); a central interactive domain (CID) mediating self-assembly; and a C-terminal GTPase effector domain (GED) (containing leucine zipper motif (LZ)) [143]. In recent decades, antiviral activity of Mx against a wide range of viruses has been largely reported in several fish species [144]. The first isolation of fish Mx was in perch (*Perca fluviatilis*), and the cDNA of Mx was elevated by poly(I:C) stimulation in liver [145]. In Atlantic halibut (*Hippoglossus hippoglossus*), transcription of Mx is strongly induced* in vivo* by poly(I:C) and IPNV [146]. The expression of rainbow trout Mx1, Mx2, and Mx3 is induced by poly(I:C) but fails to inhibit the replication of IHNV [147]. In grass carp, all the three Mx genes are induced in head kidney, spleen tissues, and CIK cells after GCRV infection; overexpression of Mxs significantly inhibits the replication of GCRV and delay the CPE induced by GCRV infection [140]. All these results demonstrate that Mx proteins are important effect molecules in host antiviral innate immunity. Interestingly, in some fish species, expression of Mx is not tightly regulated by IFN-I, although Mx is known as IFN-inducible genes. In Atlantic salmon, salmon anemia virus induces the expression of Mx through both IFN-dependent and IFN-independent ways [148]. Japanese flounder Mx induction is mediated by an IFN-independent pathway [107]. In grass carp, overexpression of Mx genes is proposed to feedback suppress expression of IFN-I [140]. Hence, the regulation of fish IFN-Mx may be more complex than that in mammals.

*(2) ISG15.* IFNs exert their antiviral effects via the induction of hundreds of ISGs. ISG15, a 15-kDa ubiquitin-like protein, is reported to be induced by IFN or viral infection. In teleost, antiviral effects of ISG15 have been reported in Atlantic salmon [149], Atlantic cod (*Gadus morhua*) [150, 151], tongue sole (*Cynoglossus semilaevis*) [152], orange-spotted grouper [153]. Particularly, in cyprinid fish, ISG15 was proposed to exert antiviral activity against both RNA and DNA viruses in zebrafish [154]; crucian carp ISG15 have two homologues and both of them were induced by GCHV and poly(I:C) challenge [155].

*(3) PKR and PKZ.* dsRNA dependent protein kinase (PKR) and Z-DNA binding protein kinase (PKZ) play an important role in the innate immune response against viral infection. Fish PKR has a dsRNA binding domain (dsRBD) (containing two dsRNA binding motifs) and an eIF2*α* kinase domain at the N- and C-terminal, respectively. Serving as orthologs of PKR, PKZ contains two left-handed deoxyribonucleic acid (Z-DNA) binding domians (ZBDs) instead of dsRBD of PKR [156]. Fish PKR and PKZ genes show similar genomic organization [157]. In cyprinid fish, both grass carp PKR and PKR and rare minnow PKZ are expressed ubiquitously at a low-level in various tissues [158–160]. Upon GCRV infection, rare minnow PKZ and grass carp PKR are significantly upregulated; the expression of grass carp PKZ is also increased by poly(I:C) stimulation [158–160]. These results may provide evidence that fish PKR and PKZ is involved in antiviral immune response to dsRNA infection.

*(4) Gigs.* GCRV-induced genes 1 and 2 (Gig1 and Gig2) are first identified as novel fish ISG from UV-inactivated GCRV-infected crucian carp blastulae embryonic (CAB) cells [161]. GCRV infection induces expression of both Gig1 and Gig2 via newly synthesized CBA IFN [161]. Further studies indicate that crucian carp Gig1 can be induced by poly(I:C) through RIG-I-triggered IFN signaling pathway; and the expression of Gig2 is dependent of IRF7 upon poly(I:C) or IFN stimulation [162, 163]. Overexpression of zebrafish Gig2 can protect cultured fish cells from virus infection [164]. However, in grass carp, both Gig1 and Gig2 expression can be induced by GCRV but not by recombinant grass carp IFN [165]. So grass carp Gigs induction may be in an IFN-independent pathway.

*(5) Viperin.* Viperin is a typical IFN-induced antiviral protein in mammals. Fish viperin has been identified in many species and proposed to establish an antiviral state in early antiviral response [7]. Overexpression of crucian carp viperin confers significant protection against GCRV infection, which is through RLR-triggered IFN signaling pathway [141].

*(6) ADAR1.* Adenosine deaminase acting on RNA (ADAR) is an RNA editing enzyme that targets both coding and noncoding dsRNA. Three ADARs (ADAR1, ADAR2, and ADAR3) are present in mammals, and there are two protein size forms (p110, p150) of ADAR1, and ADAR1 p150 is IFN-inducible protein [166, 167]. Evidence indicates that ADAR is capable of both antiviral and proviral dependent on the type of viruses [166]. In grass carp, transcript of ADAR was upregulated by GCRV and poly(I:C)* in vivo* or* in vitro* [168].

#### 3.6.3. TRIMs

The tripartite motif (TRIM) proteins recently emerged as novel mediators in antiviral immunity [169]. The TRIM proteins are characterized by a tripartite motif that consists of a N-terminal RING zinc finger domain, one or two B-box domains, and a C-terminal coiled coil domain [170]. The RING domains of TRIMs confer E3 ubiquitin ligase activity which allows TRIM to mediate ubiquitylation event; the B-box domains have been shown to contribute to innate resistance to HIV; and the C-terminal domains are involved in specific interactions and cellular localization [170, 171]. To date, more than 77 TRIMs have been identified in human [171]. Additionally, alternative splicing from a TRIM gene forms multiple TRIM transcripts [169]. Fish TRIM family has been subjected to a quick, extensive diversification by duplication and specialization. Report describes 84 fish novel TRIM proteins named finTRIMs in zebrafish [172].

Recently, studies highlight the positive or negative roles of TRIM in innate immune response preventing or curtailing pathogen invasion [170, 173]. Multiple TRIM members involve in antiviral immunity at various levels of the IFN signaling cascade [170, 174]: some TRIMs are IFN inducible and restrict viral infection such as TRIM5, TRIM8, and TRIM22 [175–177], while some TRIMs expressions mediate the production of IFN such as TRIM25, TRIM21, and TRIM68 [174, 178–180]. This is why researchers classify TRIMs into ISG families [7, 142]. However, recent studies reveal multifaceted feature of TRIMs in innate system. Evidences propose that TRIM proteins are involved in the regulation of PRRs pathways. TRIM25 RING finger E3 ubiquitin ligase that induces robust ubiquitination of CARD of RIG-I is essential for RIG-I-mediated antiviral activity [178]. Like TRIM25, TRIM4 interacts with the CARD of RIG-I and targets the K63-linked ubiquitination and regulates the virus-induced IFN induction [181]. TRIM59 suppresses RLR-induced activation of IRFs and NF-*κ*B via interaction with evolutionarily conserved signaling intermediate in Toll pathways (ECSIT) [182]. TRIM32 modulates IFN-I induction and cellular antiviral response by targeting MITA for K63-linked ubiquitination [183]. TRIM38 negatively regulates TLR3-mediated IFN-I signaling by targeting TRIF for degradation [184]. Studies also indicate that TRIM38 negatively mediates TLR3/4- and RIG-I-mediated IFN-*β* production and antiviral response by interacting with NF-*κ*B-activating kinase associated protein 1 (NAP1) and inhibits TLR-induced activation of NF-*κ*B and MAPK by targeting TRAF6 [185, 186]. TRIM27 negatively regulates NOD2-mediated signaling by degradation of NOD2 [187].

At present, more and more evidences underline the mechanisms of TRIM family proteins in restricting viral infection. However, the diversity, splicing variants, and differences in tissue expression and subcellular localization decide the versatility and complexity of TRIMs. In fish, the biological function of TRIMs is rarely understood. By deep sequencing, a large number of TRIMs were upregulated in adult rainbow trout upon viral stimulation, which suggests TRIMs are involved in antiviral immunity [169].

#### 3.6.4. Other Immune Genes Involved in GCRV Infection

Besides classical PRRs network and ISGs, some other genes are also participating in immune defense against GCRV, such as GCRV receptor junction adhesion molecule A (JAM-A) [188], voltage-dependent anion-selective channel proteins (VDACs) [189], ubiquitination pathway-related Nedd4 binding protein 1 (N4BP1) [190], lipopolysaccharide-induced TNF-*α* factor (LITAF) [191], T-bet, and GATA-3 [192].

## 4. Molecular Genetic Markers

Genetic markers evolve from phenotype marker to molecule marker with the development of the biotechnology. Molecule markers based on PCR technique which provides precise and rapid varietal identification are widely used in genotyping, breeding, and genetic studies [193, 194]. Up to date, some molecular marker technologies have been used in fish species. The markers include restriction fragment length polymorphism (RFLP), amplified fragment length polymorphism (AFLP), sequence-related amplified polymorphism (SRAP), random amplified polymorphism DNA (RAPD), simple sequence repeats (SSR, also known as microsatellite), single nucleotide polymorphism (SNP), and mtDNA and insertion/deletion (InDel) [17, 195–197]. Among these markers, SNP and SSR are the two most utilized [198]. The polymorphisms of SNP and SSR are generated by different mechanisms: SNPs are single base pair substitutions distributed throughout the nuclear genome, while SSRs are short stretches of nuclear DNA composed of a motif repeated *n* times, centered between less repetitive flanking regions [198].

In grass carp, both SNP and SSR techniques were used [17, 199, 200]. Particularly, some SNPs associated with resistance/susceptibility to GCRV were identified in some important antiviral immune genes such as TLR3 [17], TLR22 [58], RIG-I [101], MDA5 [100], LGP2 [102], and IPS-1 [16]. These polymorphisms may provide some precious information for further research of disease resistance traits and genetic breeding of grass carp. However, most SNPs are thought to be biallelic and have lower information content [201]. So more advanced technology or multiple genetic marker techniques need to be conjunctively utilized to screen useful molecular marker.

## 5. Prevention and Treatment

### 5.1. GCRV Detection

So far, many methods have been developed to detect GCRV, such as virus isolation, electron microscopy observation, antigen-related serological reactions, genome-related nucleic acid hybridization, and RT-PCR techniques which permit the detection of GCRV in an easy, fast, and efficient way [202]. Recently, the reverse transcription loop-mediated isothermal amplification (RT-LAMP) has been used for GCRV diagnosis, which is rapid, easy and no complicated instrument required is used for GCRV diagnosis [203]. Furthermore, antigenic serodiagnosis using antibody of grass carp IgM is also employed for GCRV detection [204–206].

### 5.2. Preventive and Therapeutic Strategies for Hemorrhagic Disease Control

#### 5.2.1. Vaccine

Among different disease management strategies, vaccination has proved to be a very effective way of protecting fish from viral disease [207]. Fish viral vaccines have gone through three stages: the first stage of inactivated vaccine and attenuated vaccine; the second stage of recombinant subunit vaccine; and the third stage of DNA vaccine (also known as nucleic acid vaccine or genetic vaccine) [207, 208]. In China, the first vaccine for grass carp hemorrhagic disease “organization plasma inactivated vaccine” was obtained in 1960s. Subsequently, significant achievements have been obtained in inactivated vaccine and attenuated live vaccine through cell culture for hemorrhagic disease of grass carp [209, 210]. In 2011, a live vaccine for GCRV-892 strain developed by the “Pearl River Fishery Research Institute, Chinese Academy of Fishery Sciences” obtained the “State Medicine Manufacturing Approval Number” awarded by the pharmaceutical supervisory and administrative department of the State Council of People's Republic of China, which is the first State Medicine Manufacturing Approval Number in vaccine for aquatic animals in China. This vaccine is widely applied nowadays. Recombinant subunit vaccine uses recombinant specific viral proteins (viral subunits) as antigens. Research has reported that antibodies against GCRV outer-capsid proteins VP5 and VP7 expressed in* E. coli* can neutralize viral infectivity [211]. Recombinant VP4 of GCRV-GD108 can also induce strong immune response [212]. However, this vaccine is easily degraded during processing, delivery or in the animals [207]. To data, study of recombinant subunit vaccine is still only experimental.

DNA vaccine is an organism with naked DNA representing a viral encoded protein which is under the control of a strong promoter. By intramuscular injection or gene gun bombardment of the epidermis, the naked DNA expresses recombinant viral antigen protein using the host encoding system, which further induces the host immune defense [207]. For aquatic organisms, DNA vaccines offer several advantages over the classical antigen vaccines, such as inexpensive, stable, and easy to produce, modify, and store [208, 213]. These advantages catch widespread attention of scientists. Recent years, DNA vaccines of grass carp hemorrhage have achieved gratifying progress. Studies have demonstrated the immunogenicity of GCRV structural proteins such as VP4 [206], VP6 [214], and VP7 [204, 215]. DNA vaccine of GCRV VP6 gene has been provided with significantly protective effect against hemorrhagic disease [216]. It is believed that in the coming years DNA vaccines will play a vital role in the prevention of grass carp hemorrhagic disease.

#### 5.2.2. RNAi

RNAi is a highly conserved gene-silencing mechanism caused by dsRNA in both plants and animals [217]. RNAi-mediated virus suppression is dependent on Dicer, an RNAse III type endonuclease, which inhibits viral replication via recognition of viral dsRNA or structured RNA, and initiation of RNA-based viral immunity [218]. Grass carp Dicer has been identified and can be induced by GCRV infection both* in vitro* and* in vivo* [219]. The genomic dsRNA of GCRV is sensitive to the cellular RNAi pathway, which sheds light on the interaction between RNAi antiviral pathway and aquareovirus infection [220]. Previous study has demonstrated that RNAi technique can suppress the replication of GCRV [221].

#### 5.2.3. Preventive Medicines

With the deficiency of traditional therapy, novel antireovirus strategies have been developed according to the pathogenesis of GCRV. (1) Protease inhibitor: protease inhibitor can prevent viral nucleic acid decladding from GCRV outer capsid, which can inhibit GCRV infection in host cells; (2) IFN inducer: IFN-mediated immune response is the main pathway against GCRV infection. IFN inducer can facilitate IFN production that activates some kinases and induces antiviral immune response. (3) GCRV interfering particles: adding virus interfering particles can reduce the infectivity of progeny virus, which further suppress virus proliferation [218]. (4) Medicines such as mycophenolic acid, chestnut, and quebracho woods are proposed to specifically inhibit GCRV [222, 223].

## 6. Perspective

Recently, great progresses on antiviral immunity have been achieved in teleosts. Many mammalian antiviral genes have been characterized, and the functions are investigated in some model or economic fish species. Some fish specific immune genes were also identified. However, studies on the regulation mechanisms of fish antiviral immune signaling pathways remain far behind those of mammals. The abundant alternative splicing and gene duplication present in fish make fish innate immunity more complicated. With species diversity, differences in research methods and unbalanced development between different species and contradictory or confused results often come from different studies on the same genes. Fortunately, the wide application of high-throughput sequencing allows the finding of novel antiviral-related genes more rapidly and precisely. However, researches on translation level are urgent to investigate the precise regulation mechanism of immune pathway such as ligand recognition or protein interaction. As for GCRV, complete genome sequence anatomy and functional investigation of capsid proteins lay a foundation for the development of antireovirus strategies. Application of existing vaccine technology and other therapeutic methods effectively prevent virus infection. However, more effort is needed to achieve large-scale factory application. For animal disease, prevention is better than cure. Our short-term goal is to exploit novel preventive medicines and vaccines. However, in the long run, uncovering the gene regulation network relative to virus disease resistance, identifying functional genes and molecular markers, and developing novel materials and technologies for molecular breeding are the main issues for us.

## Figures and Tables

**Table 1:** The known GCRV strains, sequences, and the corresponding GenBank accession numbers.

GCRV	Segments or genes	GenBank numbers
GCRV-873	S1–S11	AF260511, AF260512, AF260513, AF403390, AF403391, AF403392, AF403393, AF403394, AF403395, AF403396, AF403397
AGCRV	S1–S11	NC_010584, NC_010585, NC_010586, NC_010587, NC_010588, NC_010589, NC_010590, NC_010591, NC_010592, NC_010593, NC_010594
GCRV-HZ08	S1–S11	GQ896334, GQ896335, GQ896336, GQ896337, GU350742, GU350743, GU350744, GU350745, GU350746, GU350747, GU350748
GCRV-HuNan794	S1–S11	KC238676, KC238677, KC238678, KC238679, KC238680, KC238681, KC238682, KC238683, KC238684, KC238685, KC238686
GCRV106	S1–S11	KC201166, KC201167, KC201168, KC201169, KC201170, KC201171, KC201172, KC201173, KC201174, KC201175, KC201176
GCRV-GD108	L1, L2, L3, M4, M5, M6, S7, S8, S9, S10, S11	HQ231198, HQ231199, HQ231200, HQ231201, HQ231202, HQ231208, HQ231203, HQ231204, HQ231205, HQ231206, HQ231207
GCRV918	S1–S11	KC201177, KC201178, KC201179, KC201180, KC201181, KC201182, KC201183, KC201184, KC201185, KC201186, KC201187
HGDRV (GCRV104)	S1–S11	JN967629, JN967630, JN967631, JN967632, JN967633, JN967634, JN967635, JN967636, JN967637, JN967638, JN967639
GCReV-109	S1–S11	KF712475, KF712476, KF712477, KF712478, KF712479, KF712480, KF712481, KF712482, KF712483, KF712484, KF712485
GCRV-HeNan988	S1–S11	KC847320, KC847321, KC847322, KC847323, KC847324, KC847325, KC847326, KC847327, KC847328, KC847329, KC847330
GCRV096	S6, S10	JN206664 [27], JN206665
GCRV 097	S3 (VP3), S5 (VP5), S6 (VP4), S8 (VP41)	JQ782741, JQ782742, JQ782743, GQ469997
GCRV-991	S8 (VP6), S10 (VP7)	AF403414, AF403411
GCRV-875	S8 (VP6), S10 (VP7)	AF403412, AF403409
GCRV876	S8 (VP6), S10 (VP7)	AF403413, AF403410
GCRV-JX01	VP4, NS38, VP7	JQ042805, JQ042807, JQ042806
GCRV-JX02	S10, VP11	JX263303, JQ042808
GCRV-ZS11	S9 (VP6)	KC130082
GCRV-YX11	S9 (VP6)	KC130081
GCRV-JS12	S9 (VP6)	KC130077
GCRV-NC11	S9 (VP6)	KC130078
GCRV-QC11	S9 (VP6)	KC130079
GCRV-QY12	S9 (VP6)	KC130080
GCRV-HS11	S9 (VP6)	KC130076
GCRV-HN12	S9 (VP6)	KC130075
